# Supplementary material for: The Impact of Swallowing Difficulties on Quality of Life in Adults with Intellectual Disabilities in Residential Care: Cross-Sectional Study
Source: Int J Environ Res Public Health. 2025 Sep 24;22(10):1470. doi: 10.3390/ijerph22101470 (PMC12563809; doi:10.3390/ijerph22101470)
Supplement: Supplementary file 1 [file ijerph-22-01470-s001.zip › ijerph-3804305-supplementary.pdf]

Table S1. Spearman's rank correlation coefficients among candidate variables for the generalized linear model

	EAT-10	Age	FIM	Barthel Index	BMI	Fat mass	MNA-SF®	Hand grip	Number of teeth
EAT-10	1	0.292*	−0.478**	−0.551**	−0.227	−0.245	−0.356*	−0.487**	−0.421**
Age		1	−0.036	−0.183	−0.148	0.028	−0.266	0.002	−0.643**
FIM			1	0.909**	0.096	0.180	0.277	0.186	−0.006
Barthel Index				1	0.205	0.298*	0.345*	0.226	0.088
BMI					1	0.892**	0.751**	0.142	0.080
Fat mass						1	0.723**	−0.007	−0.072
MNA-SF®							1	−0.013	0.120
Hand grip								1	0.019
Number of teeth									1

Table S2. Sensitivity analysis using generalized linear model with EAT-10 as a continuous variable

Variables	B ± SE	95% Wald CI [a]	*p*-value
EAT-10	−0.05 ±0 .01	−0.06-−0.03	<0.001
Age	−0.01 ± 0	−0.01-0	0.047
Gender Female	−0.28 ± 0.06	−0.39-−0.17	<0.001
Male	reference		
Number of teeth	−0.01 ± 0	−0.02-0	0.005
Barthel Index	0 ± 0	0-0	0.525
Hand grip	0.01 ± 0.01	0-0.02	0.041
MNA-SF®	−0.03 ± 0.02	−0.06-0.01	0.177

Generalized linear model with gamma distribution and identity link function. The analysis examines the dose–response relationship between swallowing dysfunction and EQ-5D.

a: confidence interval

The generalized linear model significantly improved model fit over the intercept-only model (likelihood ratio $\chi^2(7) = 33.7$, $p < .001$)

$R^2 = 0.454$, adjusted $R^2 = 0.317$; variable-level contribution for EAT-10: partial $\eta^2 = 0.198$.
